# Enzyme-triggered- and tumor-targeted delivery with tunable, methacrylated poly(ethylene glycols) and hyaluronic acid hybrid nanogels

**DOI:** 10.1080/10717544.2022.2105443

**Published:** 2022-08-07

**Authors:** Wioletta Liwinska, Ewelina Waleka-Bagiel, Zbigniew Stojek, Marcin Karbarz, Ewelina Zabost

**Affiliations:** a Faculty of Chemistry, Biological and Chemical Research Centre, University of Warsaw, Warsaw, PL, Poland; b Faculty of Chemistry, Warsaw University of Technology, Warsaw, PL, Poland

**Keywords:** Targeted drug delivery, enzymatic degradation, controlled release, hybrid network nanogel, methacrylated hyaluronic acid, poly(ethylene glycol)

## Abstract

Enzyme-responsive polymeric-based nanostructures are potential candidates for serving as key materials in targeted drug delivery carriers. However, the major risk in their prolonged application is fast disassembling of the short-lived polymeric-based structures. Another disadvantage is the limited accessibility of the enzyme to the moieties that are located inside the network. Here, we report on a modified environmentally responsive and enzymatically cleavable nanogel carrier that contains a hybrid network. A properly adjusted volume phase transition (VPT) temperature allowed independent shrinking of a) poly(ethylene glycol) methyl ether methacrylate (OEGMA) with di(ethylene glycol) and b) methyl ether methacrylate (MEO_2_MA) part of the network, and the exposition of hyaluronic acid methacrylate (MeHa) network based carboxylic groups for its targeted action with the cellular based receptors. This effect was substantial after raising temperature in typical hyperthermia-based treatment therapies. Additionally, novel tunable NGs gained an opportunity to store- and to efficient-enzyme-triggered release relatively low but highly therapeutic doses of doxorubicin (DOX) and mitoxantrone (MTX). The controlled enzymatic degradation of NGs could be enhanced by introducing more hyaluronidase enzyme (HAdase), that is usually overexpressed in cancer environments. MTT assay results revealed effective cytotoxic activity of the NGs against the human MCF-7 breast cancer cells, the A278 ovarian cancer cells and also cytocompatibility against the MCF-10A and HOF healthy cells. The obtained tunable, hybrid network NGs might be used as a useful platform for programmed delivery of other pharmaceuticals and diagnostics in therapeutic applications.

## 1. Introduction

Despite many advances in diagnosis and curing of cancer, the evolution of various cancer signaling pathways and effective targeted pharmacological treatments remain the research challenge today (Ma et al., 2022; Mei et al., 2022). Among various proposed ways of targeted cancer treatment, those using the nanopolymer-based vehicles are highly promising in practical applications (Min et al., 2015; Mi et al., 2020). Typical polymer-based delivery objects can have the form of micelles, capsules, and generally micro-, and nanogels (Zhao et al., 2018; Preman et al., 2021). Core-shell-based structures were also reported (Amir & Khatoon, 2019). Notably, the application of targeted nanogels allowed the treatment of intractable brain tumors and multi-drug resistant- (MDR), metastatic- and relapsed tumors (Furtado et al., 2018; Mei et al., 2022; Yan et al., 2022).

Nanogels (NGs) are highly internally crosslinked polymeric hydrogels, often co-polymerized. NGs sizes range from 20 nm to 200 nm, which allows them to escape by renal clearance. Typically, nanogels with a diameter of 100–200 nm circulate in blood longer (Min et al., 2015). Their tridimensional interior hydrophilic networks offer water- and physiological fluid retaining properties, colloidal stability, large surface area, tunability, and prevention of dimerization, and crystallization of payload chemo-drugs (Min et al., 2015; Li et al., 2019). NGs are also characterized by high stability, low viscosity, high-dose drug loading ability, longer circulation times in serum, good permeation and retention effect (EPR), programmable biodegradability, controlled and signal-triggered release, targeted delivery, nonimmunologic response, and ability to cross the blood-brain barrier (Yin et al., 2020; Ma et al., 2022).

Among proposed NGs-based solutions, a ‘smart’ controlled release and targeted delivery, gained by a combination of physical- (Liu et al., 2018; Mackiewicz et al., 2019), chemical- (Liwinska et al., 2019; Mackiewicz et al., 2021) and biomarker-based (Stanislawska et al., 2019; Massi et al., 2020) stimuli is particularly attracting. Nanogels possess a very high area-to-volume ratios, and exhibit volume phase transition (VPT) response to various external stimuli (Karbarz et al., 2017; Mollazadeh et al., 2021). Typical physical stimuli are changes in temperature, light, pH, ionic strength, and magnetic/electric field (Don et al., 2017; Wu et al., 2021). The VPT change is usually substantial and proceeds much faster in nanogels compared to regular-size hydrogels (Stanislawska et al., 2019). Essentially, an appropriate combination of stimuli-responsive crosslinkers and polymer-based networks may result in controlled, switchable and oscillating VPT response. These volume changes may result in different release times, usually referred to triggered- and prolonged delivery of drugs (Liwinska et al., 2016, 2017). It is worth noting that there are a few examples of ‘smart’ NGs, which may prove effective as clinical trials progress (Grimaudo et al., 2019; Stawicki et al., 2021).

Typical problems in passive delivery of NGs stand with its rapid neutralization by the reticuloendothelial system (RES) and enhanced permeability and retention effect (EPR). Programmed VPT related to substantial NGs size changes are being developed to exploit both effects fully. The primary strategy allows prolonged circulation of bigger NGs. They miniaturize after reaching the tumor environment due to appropriate environmental stimuli. Nanogel-mediated and tumor-selective delivery improves the membrane permeability and minimizes the activity of highly potent chemotherapeutics in off-target objects (Bertrand et al., 2014). Apart from that, the clinical usability of most biopharmaceuticals stands with its reversible tailoring to NGs networks (Cheng et al., 2018; Xu et al., 2019).

The biocompatibility and cellular internalization of NGs can be boosted by ligands with a strong affinity to overexpressed receptors or secreted proteins in the tumor microenvironment (TME) (Huai et al., 2019). Modification ways involve actively targeted lipophilic-, hydrophilic-, nucleic acids-, and peptide/protein-based strategies (Wenxing et al., 2021). The anionic polymer glycosaminoglycan, hyaluronic acid (HA), is a widely applied hydrophilic component. HA and its derivatives are pivotal in cell proliferation, invasion, and migration. HA targets two primary cell receptors: CD44 (isoforms CD44s and CD44v) and RHAMM. It also binds to LYVE1-, TLR2/4-, STAB2-, and LAYILLIN receptors (Misra et al., 2015; Spadea et al., 2019). Like many cellular factors, HA is multifunctional and has both: tumor-promoting and -suppressing properties. It should be stressed that the biological activity of HA depends on its molecular weight and the binding affinity to particular receptors. A key role in the differentiation and migration of healthy and cancer cells is the enzymatic, and non-enzymatic fragmentation, and degradation of HA. HA is degraded enzymatically by three major hyaluronidases (HYAL 1-3), primarily through hydrolysis of the β-1,4-glycosidic bonds. Catalase inhibits the degradation of HA, in a dose-dependent manner, and generates H_2_O_2_ in this reaction (Valachová et al., 2016). A non-enzymatic degradation of HA occurs in the presence of ROS (Valachová et al., 2015). The primary and rapid clearance of HA is done through the liver; the renal clearance of HA occurs as only 1% of the normal daily turnover of HA (Kim et al., 2018). The HA level and the miscellaneous ratio of high-molecular- and low-molecular-weight HA are crucial in tumor initiation, progression, recurrence, and effective treatment (Liu et al., 2019).

Poor stability and short biological half-life limit the usability of HA. HA contains carboxylic acid and hydroxyl-, and N-acetyl groups that can easily be combined with other chemicals. HA chemical modifications of carboxyl and primary alcohol groups, with either norbornene (NorHA), or methacrylate (MeHa), possess a significantly greater binding affinity to CD44. The CD44-HA interactions depend on the extent of modification, type of attacked chemical group, and the site of HA used for modification (Li et al., 2021). Amphiphilic HA derivatives can self-assemble into core-shell NGs. The internal hydrophobic core can accumulate anticancer compounds for diagnosis and treatment (Lee et al., 2012).

The majority of HA-based nanomedicines refer to their self-assembled structure (Payne et al., 2018; Cadete et al., 2019). Given HA-based NGs low immunogenicity and biodegradability, several biomedical applications have been proposed, including tissue engineering, drug delivery, and molecular imaging (Kim et al., 2019; Bayer, 2020). Several shortcomings were noticed for self-assembled forms, such as rapid degradation, poor stability, and short half-life. Considering the need to improve the functionality and performance of HA-based NGs, several ways, involving covalent modifications with the HA carboxyl-, hydroxyl-, and acetamido groups, were proposed (Valachová et al., 2016; Dovedytis et al., 2020). It is a fact that free carboxyl groups present in HA are active in targeting cellular enzymes and receptors. Such modifications allow also more prolonged circulation of NGs in the body fluids by inhibiting NGs degradation (Banerji et al., 2007). Modification paths that involve hydroxyl- and acetamido- groups achieve better amphiphilic properties, targeting and more prolonged drug release (Miao et al., 2018; Gao et al., 2019). Several excellent strategies have been proposed where a simple combination of electrostatic interactions between positive-, and negative charges of HA were employed (Li et al., 2016; Cai et al., 2019; Chen et al., 2019).

It was reported that PEG-based chemical conjugation of HA could reduce the renal clearance of protein-based drugs, reduces immune response and relieves enzyme-based degradation, thereby increases the overall tumor targeting efficacy (Leach & Schmidt, 2005). PEG-modified nanoparticles also can reduce the uptake in the liver and increase their accumulation in tumor sites (Choi et al., 2009). PEG is nontoxic, and FDA approved. One of the hot topics is the use of PEGylated HA in targeted cancer immunotherapy (Shin et al., 2017). It is known, that PEG-based NGs exhibit high levels of biocompatibility, solubility in water, and organic solvents, increased hydrophilicity, and longer times of blood circulation. We presented the anticancer activity of a new, biocompatible, and redox degradable nanogel based on p(MEO_2_MA-OEGMA), a copolymer of methyl ether methacrylate (MEO_2_MA) and poly(ethylene glycol) methyl ether methacrylate (OEGMA) (Mackiewicz et al., 2019). The appropriate weight combination of the two monomers made it possible to achieve a polymer phase transition temperature (VPT) close to body temperature (37 °C) (Duncan, 2003).

Considering NG’s stability in blood and enhanced targeting properties, we decided to synthesize a novel, enzyme-sensitive, hybrid poly(MEO_2_MA-co-OEGMA-DEGDA/MeHa-DEGDA) NGs, with a short name of MEO_2_MA-OEGMA-MeHa NGs. A simple one-pot precipitation polymerization strategy was adopted. It involved an application of di(ethylene glycol) methyl ether methacrylate (MEO_2_MA), poly(ethylene glycol) methyl ether methacrylate (OEGMA), and methacrylate hyaluronic acid (MeHa). The hybrid networks were formed by copolymerization of the above monomers with di(ethylene glycol) diacrylate (DEGDA) crosslinker. The proposed NGs possessed the ability of controlling enzymatic degradation and fragmentation. Structural degradation was correlated with the adjusted VPT temperature that was close to the body temperature. Environmental sensitiveness and triggered- and reversal VPT changes enhanced the effect of prolonged and sustained delivery. The structure of the hybrid network allowed the exposition of free carboxylic groups as nanogel shell for the encapsulation of drug and made targeted interactions with cell-based receptors possible.

## 2. Materials and methods

### 2.1. Chemicals

Poly(ethylene glycol) methyl ether methacrylate (OEGMA, average Mw = 300 g/mol), di(ethylene glycol) methyl ether methacrylate (MEO_2_MA), hyaluronic acid methacrylate (MeHA, degree of substitution: 20%-50%, MW 120,000-150,000), di(ethylene glycol) diacrylate (DEGDA 75.00%), hyaluronidase from bovine testes (Type I-S, 400-1000 units/mg, solid), potassium persulfate (KPS, 99,99%), sodium dodecyl sulfate (SDS), sodium hydroxide (NaOH), sodium chloride (NaCl), potassium chloride (KCl), monosodium phosphate (NaH_2_PO_4_) and disodium phosphate (Na_2_HPO_4_), were purchased from Sigma Aldrich (Saint Louis, Missouri, MO, USA). Doxorubicin hydrochloride (DOX) was purchased from LC Laboratories (Woburn, MA, USA). Mitoxantrone dihydrochloride (MTX) was purchased from MCE (NJ, USA). For the preparation of all solutions, deionized water (Hydrolab/HLP, Straszyn, Poland) with conductivity 0.055 μS·cm^−1^ was used.

### 2.2. Instrumentation

#### 2.2.1. Dynamic light scattering (DLS)

Determination of NGs qualitative parameters: scattered light intensity, hydrodynamic parameter (D_h_), PDI, and zeta potential (ζ), were carried out with a Malvern Zetasizer DLS instrument (Nano ZS, UK) fitted with a 4-mV He–Ne laser (λ = 632.8 nm) as the light source. The estimation of NGs hydrodynamic diameters was done with a scattering angle of 173°. For the calculation of sizes of NGs in very dilute solutions the refractive index of pure water at 25 °C (1.330) and the viscosity of water of 0.8872 cP were applied (Makra et al., 2015). A folded capillary cell with two gold electrodes was used to measure zeta (ζ) potential. Calculations of zeta potentials (ζ) from electrophoretic mobilities were done using the Smoluchowski approximation.

#### 2.2.2. Transmission electron microscopy (TEM)

The structure of the synthesized nanoparticles was observed using a Zeiss Libra 120 microscope (Oberkochen, Germany). The samples for analysis were prepared by placing 10 µL of particular nanogel solution on a formvar-coated copper grid. Then, 5 µL of 1% aqueous uranyl acetate (UA) was placed on each sample to achieve better contrast on the microscopic image. The samples were dried for 24 h.

#### 2.2.3. UV-Vis spectroscopy

UV-Vis spectra were obtained with a Thermo-Scientific spectrophotometer (Evolution 300, Waltham, MA, USA). All spectra were triply recorded in a range of wavelength 200 − 750 nm; 1-cm·quartz cuvettes were used.

### 2.3. Preparation of MEO_2_MA-OEGMA-MeHa NGs with enzymatically cleavable crosslinking

**Figure 1.**
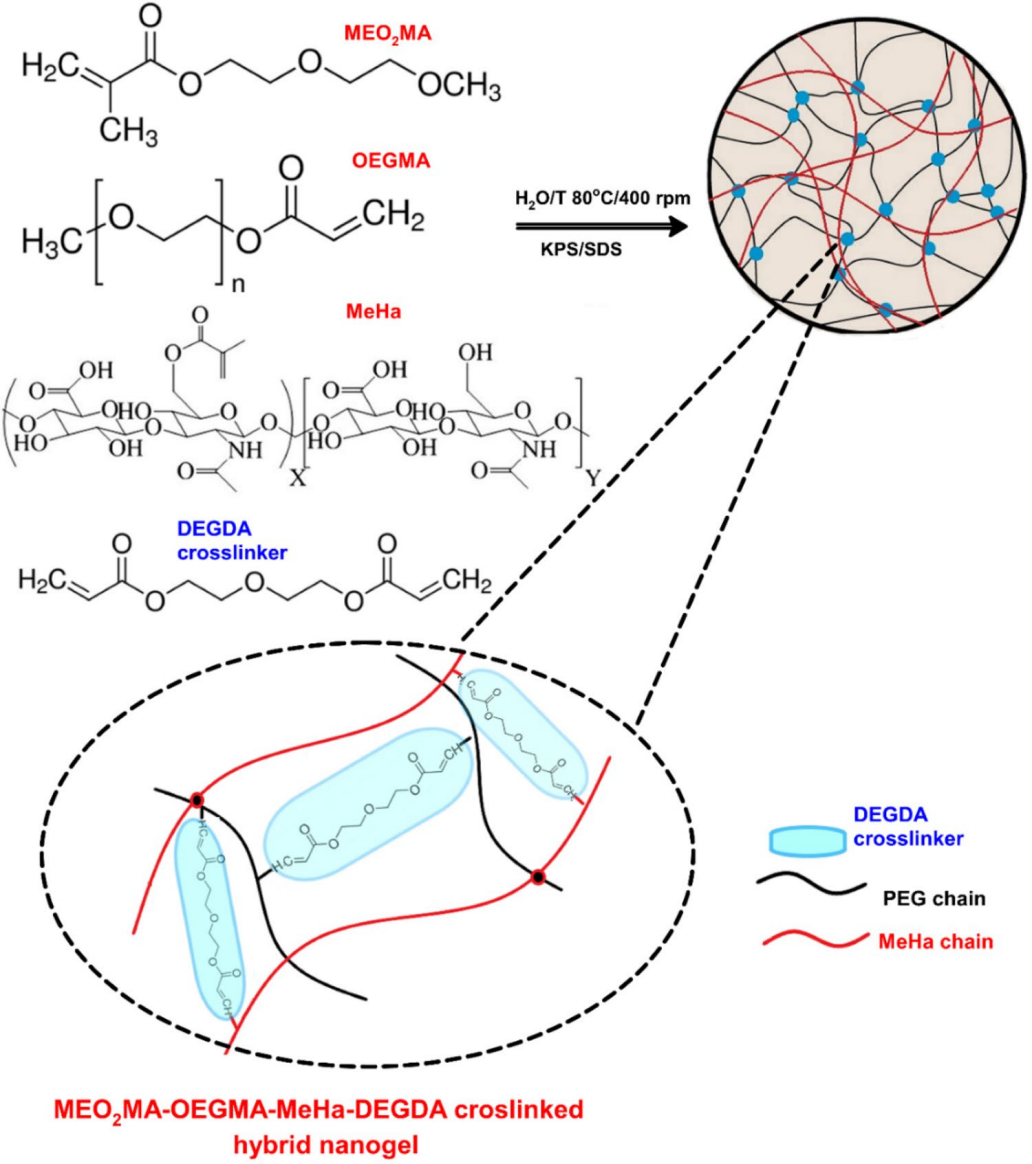
Overview of strategy in synthesis of hybrid network MEO_2_MA-OEGMA-MeHa-based NGs with enzymatically sensitive DEGDA crosslinker.

DEGDA crosslinked MEO_2_MA-OEGMA-MeHa NGs were synthesized using the precipitation polymerization method, see Figure 1 (Stanislawska et al., 2019; Ma et al., 2018). The composition of all examined NGs is listed in Table 1. All constituents were dissolved in 50 mL of deionized water with 6 mg of KPS and SDS. The total concentration of compounds equaled 64 mM. The concentration of the DEGDA crosslinker was fixed at 4 mM (mole fraction of 6%). The mole fractions of OEGMA in the solutions were 15% and 30%, respectively. The obtained mixtures were purged with argon for 1 h. Next, the solution was heated to 80 °C to initiate the polymerization reaction by thermal decomposition of KPS. Final NGs solutions were purified using a MCWO 8-10 kDa dialysis bag (Spectra/Por®, Merc, Darmstadt, Germany) and triply-distilled water. Samples were dialyzed in 5 L of water in a 7-day dialysis process at room temperature; water was changed daily. Nanogel concentration in solution, expressed as the mass of the dried nanogel, equaled circa 17 mg/ml.

**Table 1.** Composition (mM) of enzymatically sensitive hybrid network nanogels.

Sample	MeHA	DEGDA	MEO_2_MA	OEGMA
MEO_2_MA-OEGMA 15 % -MeHA-DEGDA	11.00	4.00	40.00	9.50
MEO_2_MA-OEGMA 30 % -MeHA-DEGDA	11.00	4.00	30.00	19.00
MEO_2_MA-OEGMA 30 % - DEGDA	0.00	4.00	41.00	18.50
MeHA- DEGDA	11.00	13.00	0.00	0.00

The content of the MeHA/DEGDA network is constant, and the percentage of OEGMA monomer in relation to the total concentration is 15 and 30%.

### 2.4. Enzyme-triggered degradation of NGs

For enzymatically triggered degradation of NGs, 1 mL of MEO_2_MA-OEGMA-MeHa-DEGDA NGs (17 mg/mL) was mixed with 1 mL of solution containing 2 mg/mL hyaluronidase enzyme (HAdase), 0.01 M Britton-Robinson buffer (pH = 7.4, 5.0, and 9.0). Temperature was controlled at 37 °C. The hydrodynamic parameters of prepared samples were monitored with the DLS technique.

### 2.5. Drug loading

Doxorubicin (DOX) and mitoxantrone (MTX), model anticancer drugs, were selected to study the encapsulation efficiency of the therapeutic cargo in the NGs. The drug-loading was done by subsequent mixing of 3 mL of 0.5 mM DOX and MTX solution with 3 mL of suspensions (17 mg/ml) of specific NGs. The mixture was gently stirred and kept in the dark at room temperature for 24 h. Drug-loaded NGs were separated using centrifugation (60000 r/min, 60 min). The purification process was done in dialysates, and its completeness was evaluated spectrophotometrically. Finally, the sediments of NGs were suspended in 6 mL of 0.1 M PBS of pH 7.4. Wavelength of 485 nm and 608 nm was selected for the construction of the DOX and MTX calibration curve, respectively. The R^2^ parameters of the curves were better than 0.99. Drug-loading efficiency (LE%) of the NGs were calculated using Eq. (1).

(1) $$LE=(\frac{m_{\mathrm{Dox}\;\mathrm{total}}-m_{\mathrm{Dox}\;\mathrm{free}}}{m_{\mathrm{Dox}\;\mathrm{total}}})*100\%$$

Concentration of DOX and MTX in the stocks solutions were determined from UV-Vis measurements; using extinctions coefficients (DOXε_485nm_ = 10410 L·mol^−1^·cm^−1^, MTXε_608nm_ = 19200 L·mol^−1^·cm^−1^) respectively (Tian et al., 2007).

### 2.6. pH- and enzyme triggered DOX release *in vitro*

1.5 mL of drug-loaded NGs solution with a known drug concentration were placed in a dialysis bag (MWCO = 10 kDa). The bags were dialyzed against 10 mL of 0.1 M PBS of pH 7.4 or 5.0 in the presence and absence of HAdase. The enzyme concentration was 2 mg/ml. The mixture was stirred (200 rpm) at 37 °C for 48 h. The amount of released anticancer drug was determined by measuring absorbance. The percent of cumulative drug release was calculated using Eq. (2).

(2) $$\%\mathrm{Drug}\;\mathrm{release}=(\frac{A_{t}}{A_{t0}})*100\%$$

where *A_t_* is the absorbance of the drug present in the solution at the time of particular sampling step, and *A_t0_*is the initial absorbance of the drug.

### 2.7. Cell cultures

The normal human mammary epithelial cells (MCF-10A), human breast cancer cells (MCF-7), human ovarian cancer cells (A278), and human ovarian fibroblasts (HOF) healthy cell lines were purchased from American Type Culture Collection (ATCC). MCF-7 was grown in the Dulbecco’s Modified Eagle’s Medium-DMEM (BioWest) containing 1% mixture of penicillin and streptomycin (BioWest), 1% of L-glutamine (BioWest) and 10% fetal bovine serum (FBS, Gibco). To cultivate MCF-10A the Dulbecco's Modified Eagle Medium, nutrient mixture F-12 was used. The medium was supplemented with 5% horse serum (Sigma-Aldrich), 10 ng·mL^−1^ epithelial growth factor (Sigma-Aldrich), 5 μg·mL^−1^ hydrocortisone (Sigma-Aldrich) and 10 μg·mL^−1^ human insulin (Sigma-Aldrich). All cell cultures were mycoplasma free. To cultivate the A2780 cell lines the RPMI 1640 (BioWest) containing 1% mixture of penicillin and streptomycin (BioWest), 10% fetal bovine serum (FBS, Gibco) and 1% of L-glutamine (BioWest) was used. The HOF cells were cultivated by fibroblast medium that consisted of 500 ml of basal medium, 10 ml of fetal bovine serum (FBS), 5 ml of fibroblasts growth supplement (FGS), and 5 ml of antibiotic solution (P/S).

### 2.8. Cell viability and uptake assays

The cytotoxicity abilities of DOX loaded- and pure NGs against breast cancer- (MCF-7) and healthy cell lines (MCF-10A) were investigated using the MTT assay (Waleka et al., 2020). Briefly, both cell lines were seeded onto 96- well culture plates with a density of 104 cells per well. The plates were placed in an incubator for 24 h at 37 °C under 5%-CO_2_ atmosphere to ensure cell adhesion to the well surface. Next, three solutions: DOX alone, nanogel loaded with DOX and drug-free nanogel in ranged from 0.001 µM to 10 µM were prepared. Concentrations of pure NGs corresponded to concentrations of NGs loaded with DOX. After 24 h the medium was removed from and the cells were treated with previously prepared solutions for 72 h. The control samples which consisted of the cells immersed in the appropriate medium were also prepared. After 72-h exposition, the solutions were removed and the cells were incubated with 100 µL of the MTT solution (0.5 mg·mL^−1^ in PBS). Finally, the supernatant solution was carefully removed and the formed purple crystals were dissolved in 100 µL of DMSO. To determine the viability a spectrophotometer (Cytation 3, BioTek) adapted to the work with multi-well plates was used. In addition, the influence of hyaluronidase on improvement of cytotoxicity of the tested compounds was also investigated. In this case, all tested solutions were enriched with a fixed dose of hyaluronidase enzyme present at concentration 1 mg/ml.

### 2.9. Cellular uptake by flow cytometry

The MCF-7 and A2780 cell lines were seeded onto 12-well culture plates with a density of 10^4^ cells per well. Thereafter the cells were incubated for 24 h to ensure their adhesion to the well surface. Next, the medium was removed and the cells were exposed to the DOX loaded nanogel at concentration 0.005 µM for 24 h. Concentrations of the tested compounds were measured at least 3 times. The control sample consisted of the appropriate cells immersed in the medium. Next, the cells were pulled of and centrifuged twice. The cells were suspended in the appropriate medium and analyzed use cytometer.

### 2.10. Confocal analysis

The usability of NGs-based carriers for effective transport of the drug to the cell was determined using a confocal microscope (Fluoview Olympus FV10i). The MCF7 and MCF-10A cells were seeded into a special 4-compartment cell culture dish with a density of 104 cells per well. The cells were cultured in standard conditions. Subsequently, the medium was replaced with 0.01-µM drug-loaded NGs solution. Time of experiment was corresponding to the total time of the MTT assay. The confocal imaging was done after 72 h of incubation with the drug. Before measurements the cell nuclei were stained with the Hoechst dye.

## 3. Results and discussion

### 3.1. Characterization of fine-tuned, targeted and surface active-sites NGs

**Figure 2.**
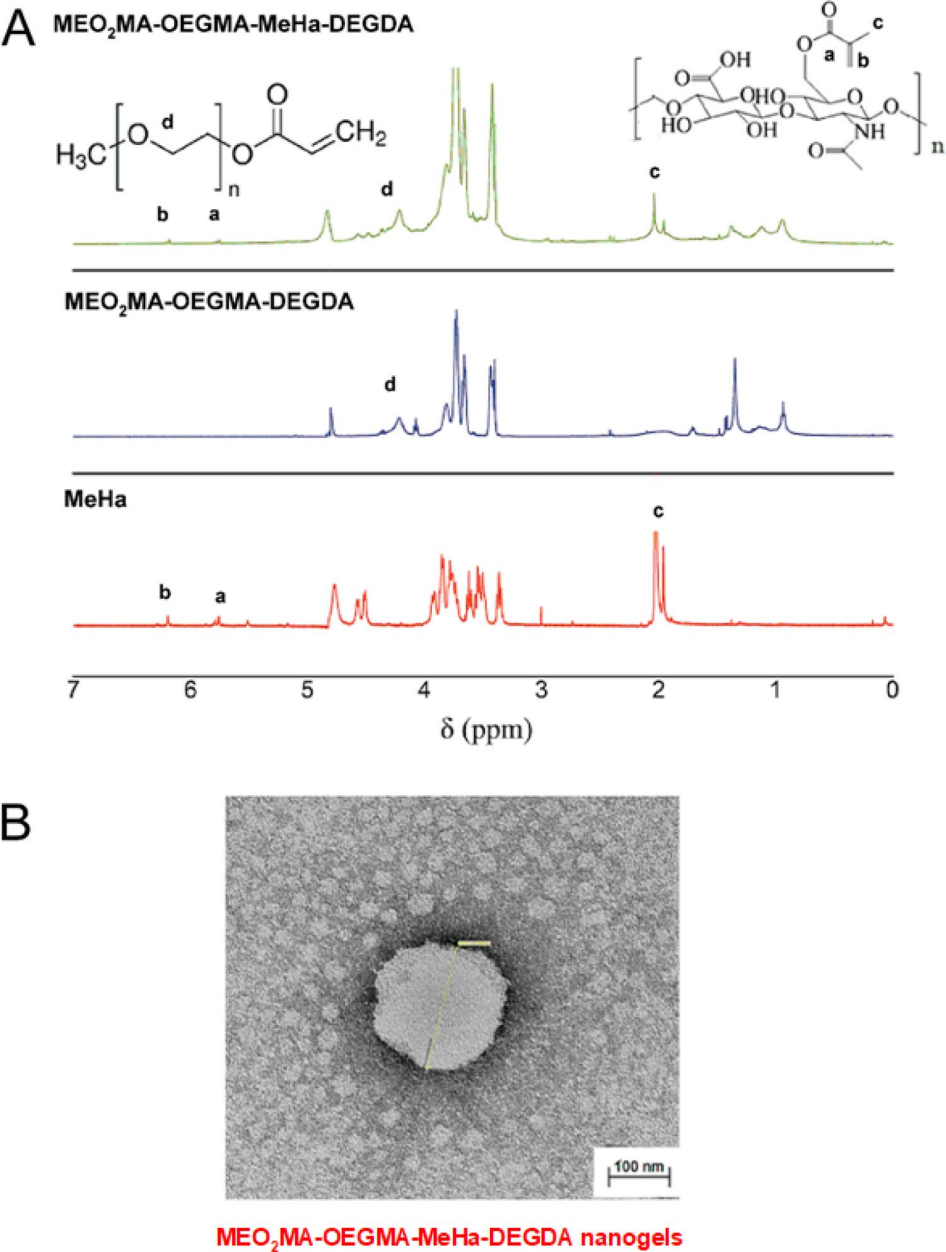
(a) Comparison of ^1^H NMR spectra of MEO_2_MA-OEGMA30%-DEGDA-, MEO_2_MA-OEGMA30%-MeHa-DEGDA NGs and MeHa polymer (in D_2_O). (b) TEM micrograph exhibiting surface activity of carboxylic groups from MeHa chains in MEO_2_MA-OEGMA30% -MeHa-DEGDA NGs obtained after application of 1% uranyl acetate (UA) staining protocol.

We synthesized a novel, hybrid network MEO_2_MA-OEGMA-MeHa copolymer to obtain the final NGs crosslinked with DEGDA with favorable properties for prolonged-, enzyme-triggered and tumor-targeting drug delivery applications, see Figure 1. Simple, one-pot precipitation polymerization strategy was developed. Successful synthesis was confirmed by comparison of H^1^ NMR spectra (25 °C) of MeHa polymer, MEO_2_MA-OEGMA-DEGDA-, and MEO_2_MA-OEGMA-MeHa NGs. All spectra are presented in Figure 2A. The DEGDA crosslinker is able to crosslink the acrylic-based polymers and the methacrylated hyaluronic acid chains. As a result, a complex hybrid structure can be obtained. The existence of hybrid network was confirmed by the presence of: a) peaks placed at 5.6- (a) and 6.1 ppm (b) corresponding to the protons referred to the methylene group and peak found at 1.8 ppm (c) related to methyl modification in the hyaluronic acid network, and b) peak found at 3.2 ppm (d) which is attributed to the three methoxy protons of the OEGMA side chain, respectively.

The morphology of MEO_2_MA-OEGMA-MeHa NGs was investigated by means of the TEM microscopy and a micrograph is presented in Figure 2B. As is it seen, the NG is spherical and has a rather irregular shape. The mean diameter of NGs was found to be circa 95 nm and the diameter range was 80 − 110 nm (25 °C, 0.1 M PBS of pH 7.4). It should be stressed, that small-scale and monodispersed NGs with lower stiffness should favor the crossing of biological barriers with focus on the blood-brain barrier (Snetkov et al., 2020). Since the gel samples were partially dried and in consequence the size of NGs was reduced by circa 30% and remained partially shrunken the state of NGs referred well to physiological conditions of healthy cells (pH 7.4, 37 °C). A better visualization of distribution of carboxylic groups in the NGs was done by staining the NGs with uranyl acetate. The picture revealed that the outer part of the NGs sphere had an improved black contrast, which indicated high density of the carboxylic groups. This observation corroborates that after the collapse of the thermosensitive MEO_2_MA-OEGMA-DEGDA part of the network at 37 °C the MeHa-based chains are exposed to the surface. That improved more efficient storing of short-time lived hyaluronic acid and its targeting action with tumor cellular receptors, as it was planned.

All determined hydrodynamic diameters (D_h_) and zeta potentials (ζ) of novel hybrid NGs, compared to single network nanogels, optimal for their prolonged blood circulation and with effective loading and releasing capacities in the enzymatically triggered cancer environment, are listed in Table 2. In particular, the appropriate weight combination of two thermoresponsive MEO_2_MA and OEGMA monomers promoted the exposition of MeHa-based carboxylic groups on the NGs surface. pH and temperature-based tunability of D_h_ was investigated by increasing component ratio in the MEO_2_MA-OEGMA-DEGDA chains in nanogels network. The obtained size of NGs was in a range from 68 to 120 nm depending on temperature in various stages of the experiment. All obtained NGs possessed a narrow dispersity (PDI < 0.115) and a slightly negative surface charge that came from the presence of active targeting carboxylic groups exposed from MeHa chains on the outer surface of the MEO_2_MA-OEGMA-MeHa NGs. The properties of double-network nanogels were clearly improved compared to single-network materials in terms of further application for the construction of drug carriers.

**Table 2.** Analysis of tunability of hydrodynamic parameters of hybrid and single network nanogels at dedicated temperatures by varying the content of thermosensitive OEGMA monomer.

Type of nanogel	T / °C	D_h_ / [nm]	ζ-potential / [mV]	**PDI** *
**MEO_2_MA-OEGMA(15%)-MeHa-DEGDA**	**25**	91.4	−7.4	0.083
	**37**	82.0	−8.4	0.111
	**45**	68.8	−9.9	0.141
**MEO_2_MA-OEGMA(30%)-MeHa-DEGDA**	**25**	121.2	−11.5	0.071
	**37**	105.7	−13.9	0.046
	**45**	94.8	−16.4	0.065
**MEO_2_MA-OEGMA(30%)-DEGDA**	**25**	121.2	−10.7	0.011
	**37**	103.9	−11.2	0.040
	**45**	99.9	−12.3	0.060
**MeHa-DEGDA**	**25**	84.4	−10.5	0.004
	**37**	83.9	−11,0	0.007
	**45**	85.5	−10.7	0.054
	**Single nanogel**		**Hybrid nanogel** **MEO_2_MA-OEGMA-MeHa-DEGDA**	
**Type of nanogel**	**MeHa-DEGDA**	**MEO_2_MA-OEGMA (30%)-DEGDA**	**15 % OEGMA (with MeHa)**	**30% OEGMA** **(with MeHa**
**MTX encapsulation efficiency** **(25 °C, pH 7.4)** **in (μM·) / in %**	100 / 40	85/ 34	70 / 28	110 / 44
**DOX encapsulation efficiency** **(25 °C, pH 7.4)** **in (μM·) / in %**				
	84 / 45	93.5 / 36	129 / 69	187 / 74.
**D_h_ [nm] / NGs with DOX, 25 °C**	98.5	145.5	109.2	135.7
**D_h_ [nm] / NGs with MTX, 25 °C**	117.5	148.6	129.6	141.5

* PDI – Polydispersity Index.

### 3.2. Modulation of NGs for an effective encapsulation/release of therapeutic cargo

**Figure 3.**
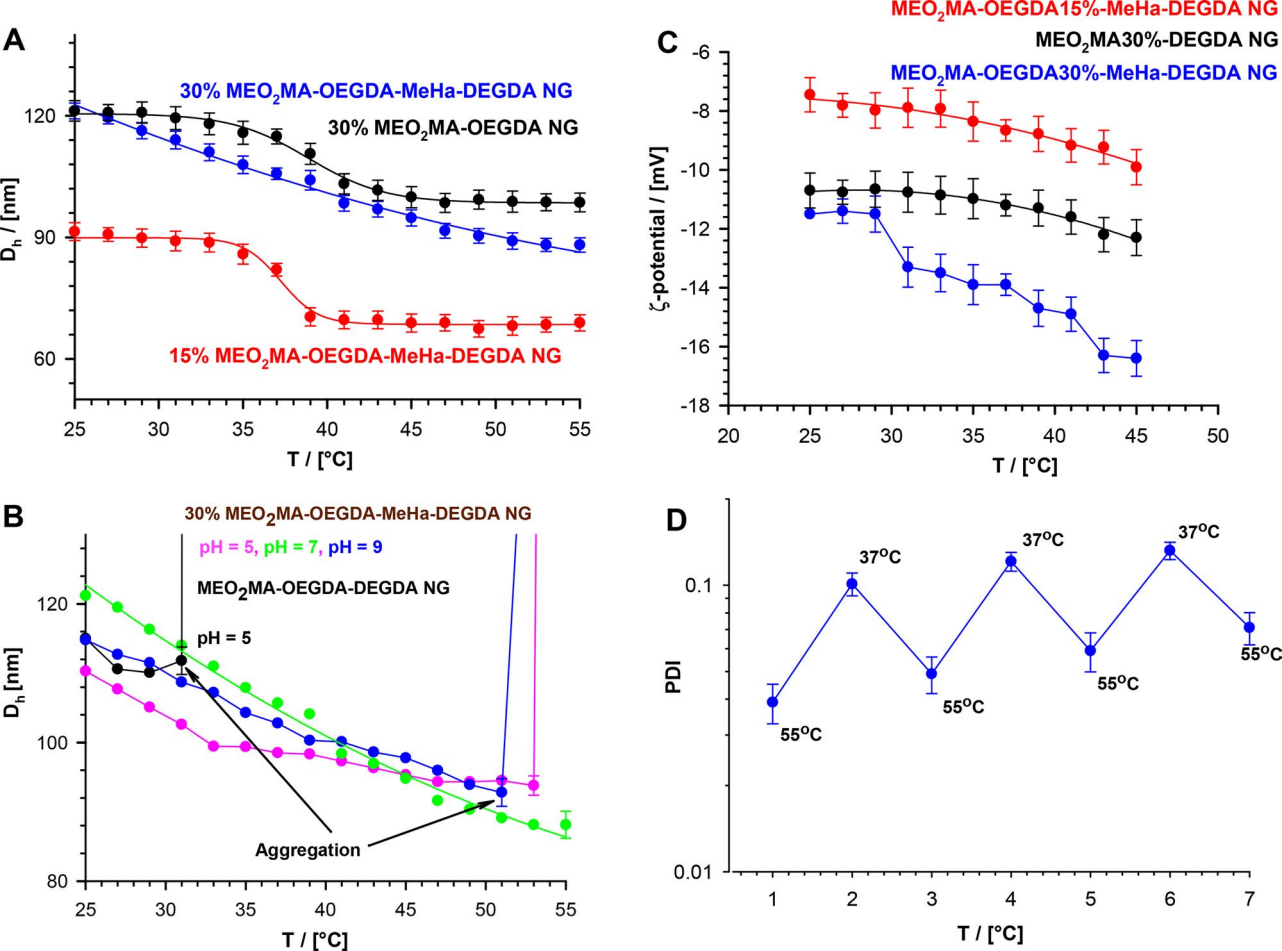
**(**a) Plots of hydrodynamic diameter, D_h_, vs temperature, obtained for MEO_2_MA-OEGDA30%-MeHa-DEGDA (blue line), MEO_2_MA-OEGDA15%-MeHa-DEGDA (red line) and MEO_2_MA-OEGDA30%-DEGDA (dark line). (b) Dependences of D_h_ on temperature obtained at various pH and referred to an aggregation process. MEO_2_MA-OEGDA- MeHa-DEGDA at pH 7.4 (green line), 5.0 (pink line), 9.0 (blue line), and MEO_2_MA-OEGDA-DEGDA at pH 5.0 (dark line). (c) Plots of zeta potential vs. temperature for increased carboxylic content in the MeHa-based network. (d) Quasi-reversible cycles of polydispersity index (PDI) changes seen at physiological- and hyperthermia-used temperatures and low acidic environment (pH 5); state of minimal tendency to NGs aggregation. All measurements were repeated three times. For clarity of the figure a single mean standard error was shown.

Temperature-responsive assembly/disassembly/aggregation of the nanogels at circa body temperature is a valuable means of controlled delivery of drugs. Modulation of the nanogel VPT temperature allows specific optimization of the encapsulation and loading cargo, that are critical as the effective drug delivery is considered. An analysis of tunability of novel environmentally sensitive MEO_2_MA-OEGMA-MeHa hybrid network NGs was done using the DLS technique. In this technique the information about the changes in the dynamics of the swollen polymeric NPs is extracted from the second-order autocorrelation function. The hydrodynamic diameter of a given particle is related to its diffusion coefficient. These parameters are linked in the Stokes-Einstein equation, Eq. (3):

(3) $$D_{h}=\frac{kT}{3\pi \eta D}$$

where *k* is Boltzmann constant, *T* is absolute temperature and *η* is solvent viscosity. In general, at pH 7.4, after raising temperature we noted a change in *ζ* and *D*_h_ which was caused by specific shrinking of the NG nets containing various amounts of the thermosensitive part of the network (MEO_2_MA-OEGMA-DEGDA), see Figure 3A and C, respectively. Both effects indicated the occurrence of dynamic redistribution of MeHa-based carboxylic groups in the outer sphere of the investigated NGs. It can be said, that the novel NGs possess a MeHa-based polymeric component that is exposed during progressive contraction of the NGs network. Certainly, the phase transition temperature of poly(ethylene glycol)-based nanogels depended on its hydrophilicity. As the content of OEGMA monomer, the longer poly(ethylene glycol) chain constituent, increased, we noticed a corresponding increase in the VPT temperature. Additionally, the presence of hydrophilic molecules of MeHa in the network structure shifted the VPT temperature to a temperature higher than the physiological one. Regarding an enzymatically-triggered and controlled degradation of NGs the MEO_2_MA-OEGMA30%-DEGDA-based network fraction of the NGs was selected for further investigations; it possessed an almost linear dependence of the hydrodynamic diameter, D_h_, on temperature. For example, at physiological temperature (37 °C) and pH 7.4 the mean D_h_ value of the MEO_2_MA-OEGMA30% -MeHa- DEGDA NGs equaled 105 ± 25 nm, while after its shrinking at a temperature of hyperthermia treatment, 55 °C, it dropped to circa 75 nm ± 38 nm. In general, despite: a) lower D_h_ and b) more effective volume phase transition of NGs with 15% MEO_2_MA-OEGMA- content, much lower aggregation were noticed for the NGs with 30% MEO_2_MA-OEGMA-based network fraction, see also Figure 3B and D, respectively. Moreover, for 30% content of MEO_2_MA-OEGMA in the NGs a two-step decrease of zeta potential, ζ, was noticed (see Figure 3C). This can be explained as the effective surface-active exposition of carboxylic groups after exposition and shrinking of the thermosensitive fraction of the NGs with profound effect after raising temperature applied in typical hyperthermia- based treatment therapies (Toraya-Brown & Fiering, 2014).

All described tunable effects were even promoted in more acidic pH in cancer cells. In general, an addition of acid or base should change the balance between the repulsive and the attractive forces between the polymer-based chains. The particles without MeHa and with 15% of the MEO_2_MA-OEGMA constituents sharply aggregated at temperatures below the physiological temperature. The exposition of MeHa chains made the MEO_2_MA-OEGMA30% -MeHa- DEGDA NGs optimally sensitive regarding the change of environmental pH from physiological to more acidic. It was noticed that these NGs shrank progressively in more acidic environment. That gave an additional opportunity to expose the MeHa-based carboxylic groups in acidic environment because pKa of HA is between 3 and 4. At a pH much higher than pK_a_ most of the carboxylic groups in the polymer network were ionized and all NGs existed in the swollen state. It should be stressed, that loading of cationic drug did not block the effective reorganization of the double-network in the NGs, and therefore the promotion of the surface-activity of the carboxylic groups was preserved. Moreover, we noticed that swelling/shrinking processes of pure and loaded NGs were reversible at 37 °C regardless the pH change from the physiological one to more acidic.

### 3.3. Enzyme-triggered degradation of nanogels

**Figure 4.**
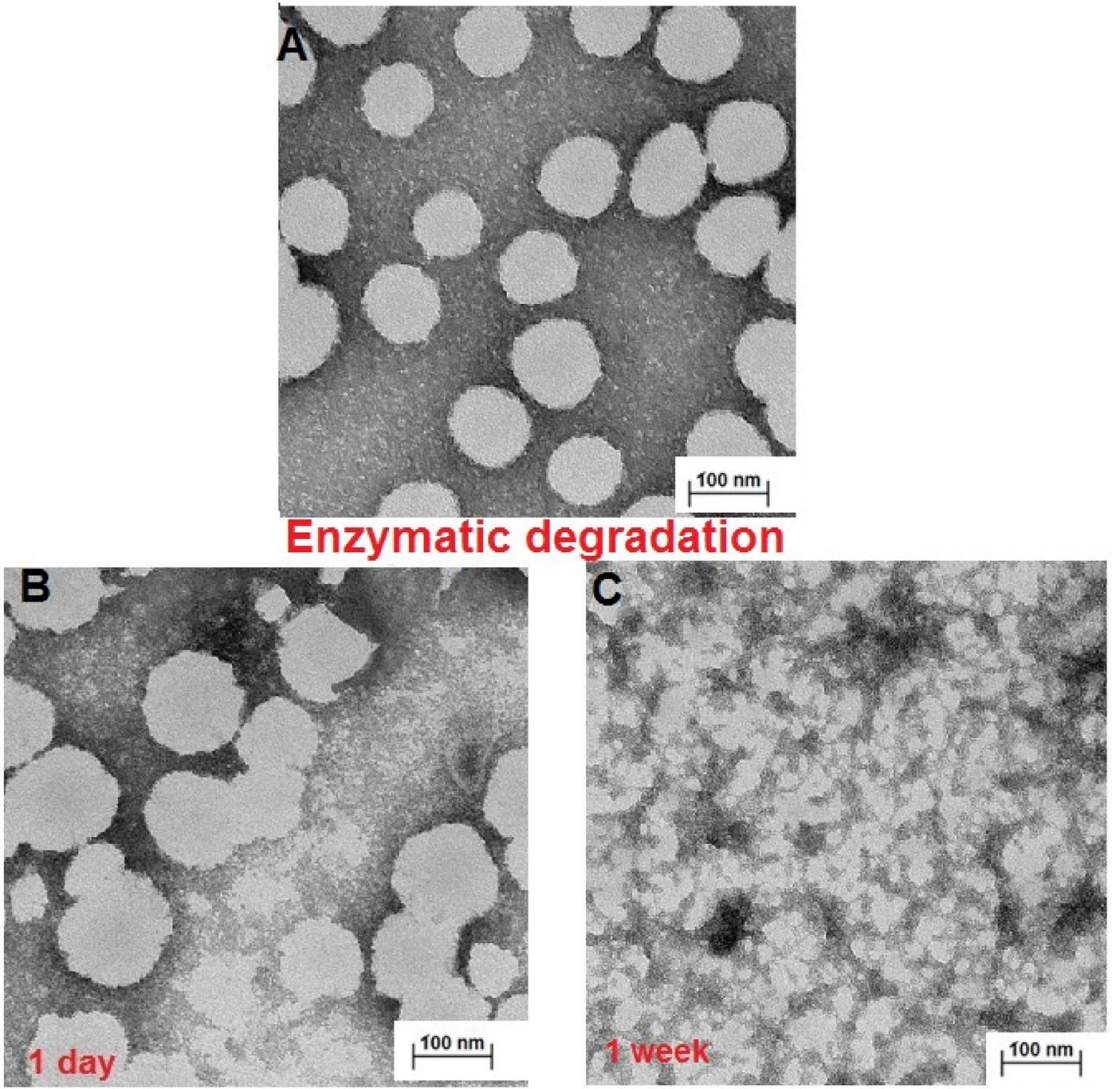
TEM micrographs of A) MEO_2_MA-OEGMA30%-MeHa-DEGDA NGs (pH 7.4), (B) MEO_2_MA-OEGMA30%-MeHa-DEGDA NGs taken 1-day after their treatment with 2 mg·mL^−1^ hyaluronidase enzyme (HAdase, pH 5.0). (C) MEO_2_MA-OEGMA30%-MeHa-DEGDA NGs reveal their HAdase-based enzymatic degradation at pH 5.0 after 1-week treatment.

The next step was to investigate the morphology of the hybrid network NGs and the products of their enzymatically- triggered (HAdase) degradation due to cleavage of ester bonds by the DEGDA crosslinker and the presence of MeHa. Figure 4 depicts TEM micrographs of examined NGs and the products of their enzymatic degradation. As it can be seen in Figure 4A, typical (MEO_2_MA-OEGMA30%-MeHa-DEGDA) NGs keep their spherical- and slightly irregular shapes; and their size distributions range from 80 to 115 nm. In general, TEM diameters agree with the D_h_ values of the partially-shrunken NGs measured at 37 °C. The progressive enzymatic degradation of nanogels was visualized after 1-day, and 1-week of its enzymatically-triggered degradation, see Figure 4B and C, respectively. Firstly, the NGs particles sizes after enzymatic degradation slightly increased, which indicated the loosening of the structure resulting from the initial degradation stage. After longer times of enzymatic exposure (1 week) a significant size reduction of up to 60% − 70% have been observed (40 nm).

**Figure 5.**
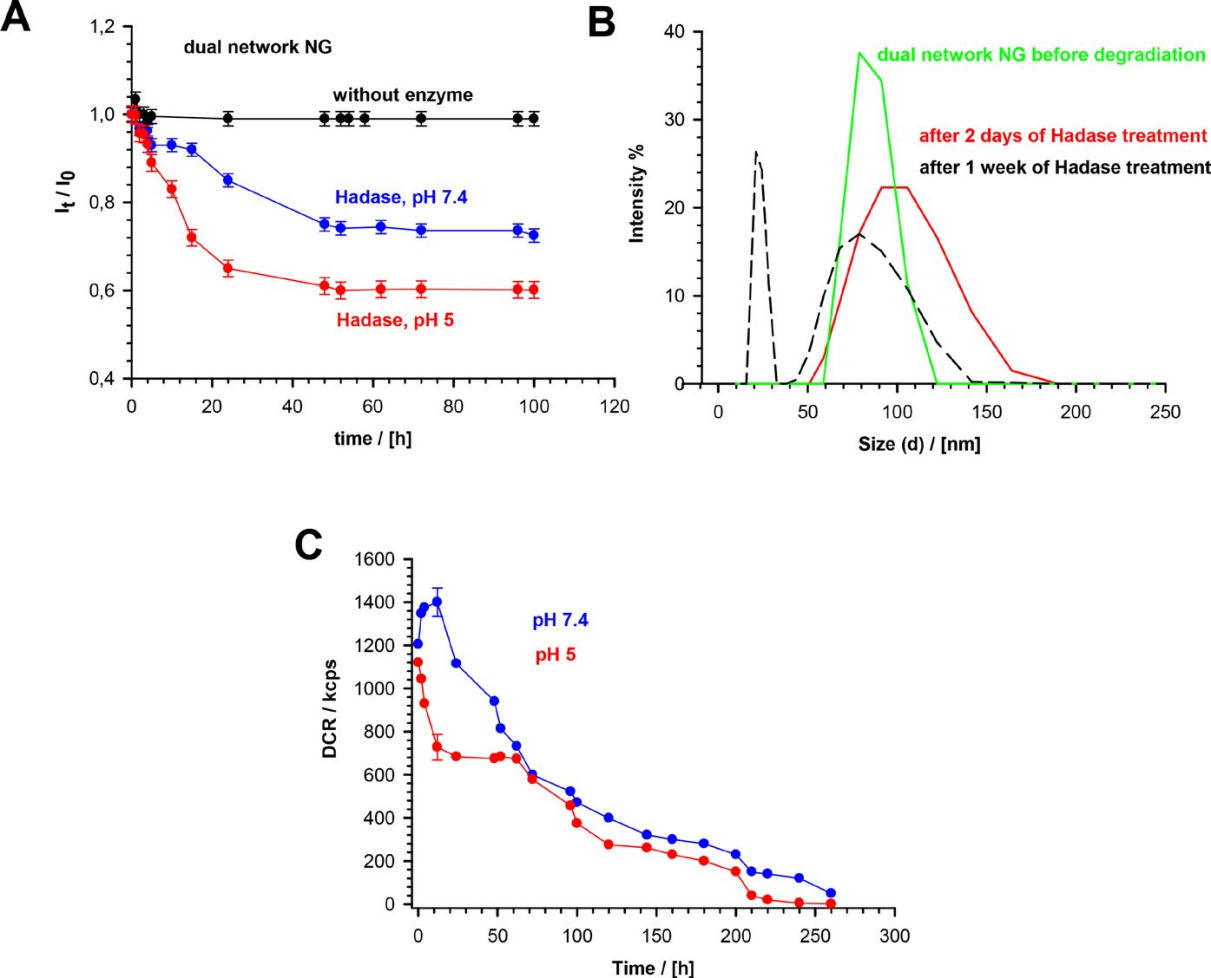
(a) Dependencies of light scattering intensity ratio, I_t_/I_o_, on degradation time of MEO_2_MA-OEGMA30%-MeHa-DEGDA NGs at 37 °C in presence and absence of HAdase. Subscripts “0” and “t” represent time t = 0 and t = t, respectively. (b) Distribution of MEO_2_MA-OEGMA30%-MeHa-DEGDA NGs size presented as changes in scattered light intensity after various treatment times by HAdase enzyme. (c) Time dependencies of scattered light intensity (derived count rate, DCR) and hydrodynamic diameter, for MEO_2_MA-OEGMA30%-MeHa-DEGDA NGs after treatment with HAdase (2 mg/ml) and at pH 7.4 and 5.0. All measurements were repeated three times. For clarity of the figure a single mean standard error was shown.

The enzymatic degradation of MEO_2_MA-OEGMA-MeHa-DEGDA NGs was quantitatively studied using the DLS technique. The changes in the directly measured nanogel light intensity, in the presence of the HAdase enzyme levels typically occurring in cancer cells (2 mg/ml), are presented in Figure 5A. Under physiological conditions (pH 7.4), the *l*_t_ parameter decreased slowly. After 20 to 40 hours a 20% decrease in the intensity was observed. In more acidic environment the light scattering intensity decreased more rapidly (40%) compared to physiological pH, so the enzymatic activity significantly increased. Figure 5B presents the distribution of NG sizes before degradation, and after 2- and 7-days of the treatment. The scattered light intensity was directly proportional to the size and number of the particles present in the sample, so we used it to estimate the extent of NGs decomposition. A 50% increase in size distribution was noticed after 2-days of HAdase treatment; the mean particle sizes increased slightly. The PDI value increased from 0.04 to 0.09, which indicated the presence of wider range of particle sizes. A decrease- and shift of the size distributions, as well as the separation of fractions of peaks after one week indicated the progressive disintegration of NGs during the enzymatic treatment. Correspondingly, the derived count rate (DCR) factor increased from 1200 to 1400 kcps during the first 3 to 4 hours of the enzymatic treatment at pH 7.4, see Figure 5C This confirmed the fact of an initial loosening of the hybrid-network of the NGs after breaking a relatively small number of the enzymatically cleaved bonds. At longer times the DCR factor started to decrease exponentially. After 140 hours of the enzymatically-triggered interaction it reached circa 200 kcps, and after 260 hours its value dropped to 50 kcps. An accelerated and multistep disintegration of NGs was observed in the first step of enzymatic degradation of NGs at pH 5.0. The DCR factor substantially decreased from 1100 to 700 kcps during the first 25 hours of the enzymatic degradation and then stabilized. After 60 h, DCR started to drop again and after 140 h it reached 210 kcps. Finally, after 260 hours it reached a value of 0.7. This specific three-step DCR change in time at pH 5.0 suggests an independent degradation of two chains in the NG hybrid network. It can be concluded, that in the first stage of an accelerated enzymatic degradation the top layer of nanoparticle formed by MeHa - DEGDA network with exposed targeted carboxylic groups undergoes a rapid disintegration. Then, two components of the polymer network located in the deeper layers of the nanoparticle disintegrate at different rates. Taking the ratio of cubes of the mean hydrodynamic diameters obtained before and after the treatment, which is a good measure of the gel degradation extent, we found that after 260 h the degradation extent reached a value of circa 95%.

### 3.4. Doxorubicin and mitoxantrone loading and release from hybrid network nanogels

The tunability and the ability to target cancer cells of the hybrid network NGs are important for the controlled release- and increased loading capacity of an anthracycline drug, doxorubicin and mitoxantrone (DOX, MTX). They are hydrophilic compounds. Therefore, the presence of the drug in the nanogel led to a slight increase, by circa 15 − 20%, in size of the NGs and raised its volume-phase-transition temperature, see Table 2. The drug loading efficiency of DOX, LE, of particular MEO_2_MA-OEGMA-DEGDA- and MEO_2_MA-OEGMA15%-MeHa- and MEO_2_MA-OEGMA30%-MeHa NGs equaled 36.4%, 69.0% and 74.1%, respectively, while loading efficiency of MTX to MEO_2_MA-OEGMA30%-MeHa NGs equaled 44%. Since pK_a_ of the amino group in the DOX and MTX molecules is reported to equal circa 8.6 and 8.3 respectively, therefore, the electrostatic interactions between the anionic carboxylic group and cationic drug could be used to load the drug into the NG hybrid network The drug loading mechanism of both drugs was similar as they were positively charged in the tested pH range The exposition of carboxylic groups on the outer layer of NGs increased the loading capacity of positively charged drug. While the zeta potential, ζ, was similar to unloaded NGs we concluded that the drug was effectively stored in the internal structure of NGs, and the ability to the surface-active targeted action of the NGs by exposed carboxylic groups took place.

**Figure 6.**
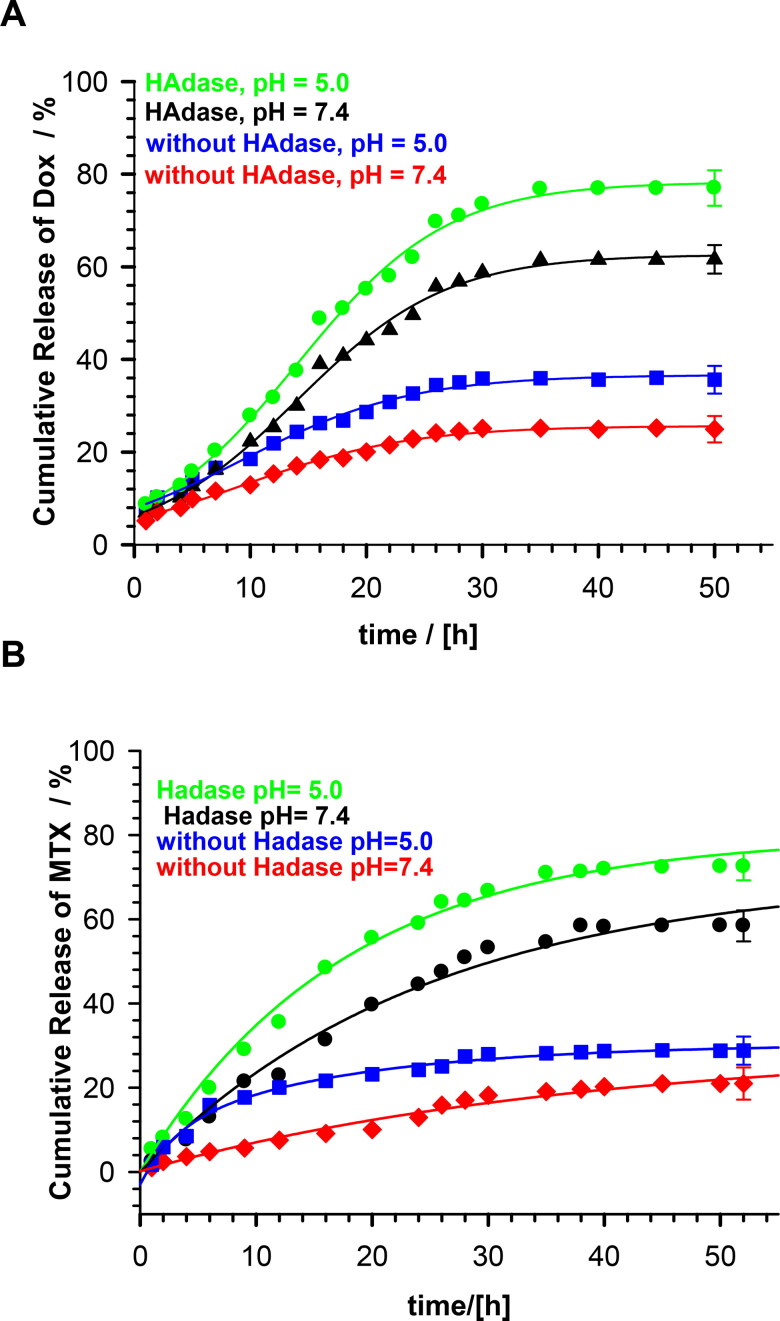
Profiles of DOX and MTX release from MEO_2_MA-OEGMA30%-MeHa-DEGDA NGs during enzymatic degradation at buffered solutions at 37 °C; HAdase concentration - 2 mg/ml. All measurements were repeated three times. For clarity of the figure a single mean standard error was shown.

The *in vitro* release profiles of DOX and MTX without- and with enzymatically triggered degradation are presented in Figure 6. Under physiological conditions (pH 7.4), the percentage of the cumulative drug release equaled circa 25% and 20% for DOX and MTX, respectively. At this pH, molecules of this drug had a positive charge and bound electrostatically to the negatively charged carboxyl groups of the nanogel, what resulted in low release. At pH 5.0, which is characteristic for cancer cells, cumulative release was circa 30% for both drugs. At this pH, more carboxylic groups were protonated and the electrostatic interactions between the nanogels and drugs were diminished. An addition of 2 mg/mL HAdase caused triggering of enzymatic degradation of the NGs. At pH 7.4 the cumulative DOX and MTX release reached 60%, while under conditions of cancer cells this value equaled 78 and 72% for DOX and MTX, respectively.

An analysis of drug release profiles in the presence of HAdase indicates sigmoidal drug release profiles. In general, if the water flux is negligible, and the swelling state of NGs before drug release is in equilibrium, the drug may be released by diffusion; 60% of the total amount of drug is released by this way. The remaining drug was released exponentially. However, drug release from the dehydrated or shrunken NG state would proceed with concurrent water uptake. Then, the swelling kinetics of gel may affect the drug release pattern. In general, we observed that DOX burst effect is limited by the state of the NGs. Clearly, DOX release profiles are influenced by the change in the water-uptake process in response to external temperature change. To describe the drug release mechanism from the synthesized NGs, different empirical models, including zero-order and first-order models, Korsmeyer–Peppas model and Higuchi model, were utilized. As it can be seen in Table 3, the equations describing kinetic models were given. It is known that the best-fit kinetic model should be selected according to the coefficient of determination. When all models are compared a significant difference can be seen. The best fitted model for release of DOX from NGs without HAdase is the Peppas model, while after the addition of HAdase it is the zero-order model. As well, the values of *k* and *n* are usually higher for NGs with HAdase. The change of the model indicates that the drug diffusion rate for NGs with HAdase treatment is much faster than the proceeding rate of the nondegraded NGs. The macromolecular relaxation rate of polymer and final NGs enzymatic degradation became rate-limiting for drug release, and therefore the zero-order release was achieved.

**Table 3.** Release parameters for MEO_2_MA-OEGMA30%-MeHa-DEGDA NGs as optimal for prolonged-, enzyme-triggered, and tumor-targeted NGs activity.

**Release conditions** *		First order	Higuchi	Korsmeyer-Peppas	Baker and Longsdale
		*F = 1 − e^−kt^*	*F = kt^0^* ^.5^	*F = kt^n^*	*F = kt*
**Release of DOX**					
**pH 7.4, HAdase**	*R^2^*	0.9731	0.9181	0.9390	0.8803
	*k*	0.039 ± 0.002	11.85 ± 0.03	8.18 ± 0.04	0.003 ± 0.0001
	*n*	–	–	0.613 ± 0.03	–
**pH 5.0, HAdase**	*R^2^*	0.9518	0.9185	0.9390	0.8934
	*k*	0.027 ± 0.001	9.48 ± 0.05	6.540 ± 0.03	0.002 ± 0.0002
	*n*	–	–	0.506 ± 0.02	–
**pH 7.4,** **without HAdase**	*R^2^*	0.5231	0.9227	0.9464	0.9425
	*k*	0.014 ± 0.02	6.08 ± 0.08	8.00 ± 0.04	0.0007 ± 0.0001
	*n*	–	–	0.416 ± 0.01	–
**pH 5.0,** **without HAdase**	*R^2^*	0.4242	0.9227	0.9465	0.9365
	*k*	0.009 ± 0.02	4.25 ± 0.05	5.60 ± 0.03	0.0003 ± 0.00002
	*n*	–	–	0.416 ± 0.01	–
**Release of MTX**					
**pH 7.4, HAdase**	*R^2^*	0.9624	0.9432	0.9455	0.9309
	*k*	0.0034 ± 0.0003	11.19 ± 0.02	9.91 ± 0.02	0.003 ± 0.0002
	*n*	–	–	0.535 ± 0.04	–
**pH 5.0, HAdase**	*R^2^*	0.9149	0.9349	0.9546	0.9149
	*k*	0.0016 ± 0.0001	8.68 ± 0.06	5.75 ± 0.04	0.001 ± 0.0003
	*n*	–	–	0.618 ± 0.03	–
**pH 7.4,** **without HAdase**	*R^2^*	0.4470	0.8906	0.9274	0.9103
	*k*	0.0093 ± 0.0004	4.69 ± 0.05	6.795 ± 0.03	0.0004 ± 0.00001
	*n*	–	–	0.392 ± 0.02	–
**pH 5.0,** **without HAdase**	*R^2^*	0.9307	0.9219	0.9652	0.9144
	*k*	0.0055 ± 0.0002	2.94 ± 0.04	5.75 ± 0.04	0.0001 ± 0.0002
	*n*	–	–	0.697 ± 0.06	–

* In all models, *F* is the fraction mass of DOX released at time *t* according to particular model, and *k* is the kinetic constant.

### 3.5. *In vitro* cytotoxicity and uptake of nanogels

**Figure 7.**
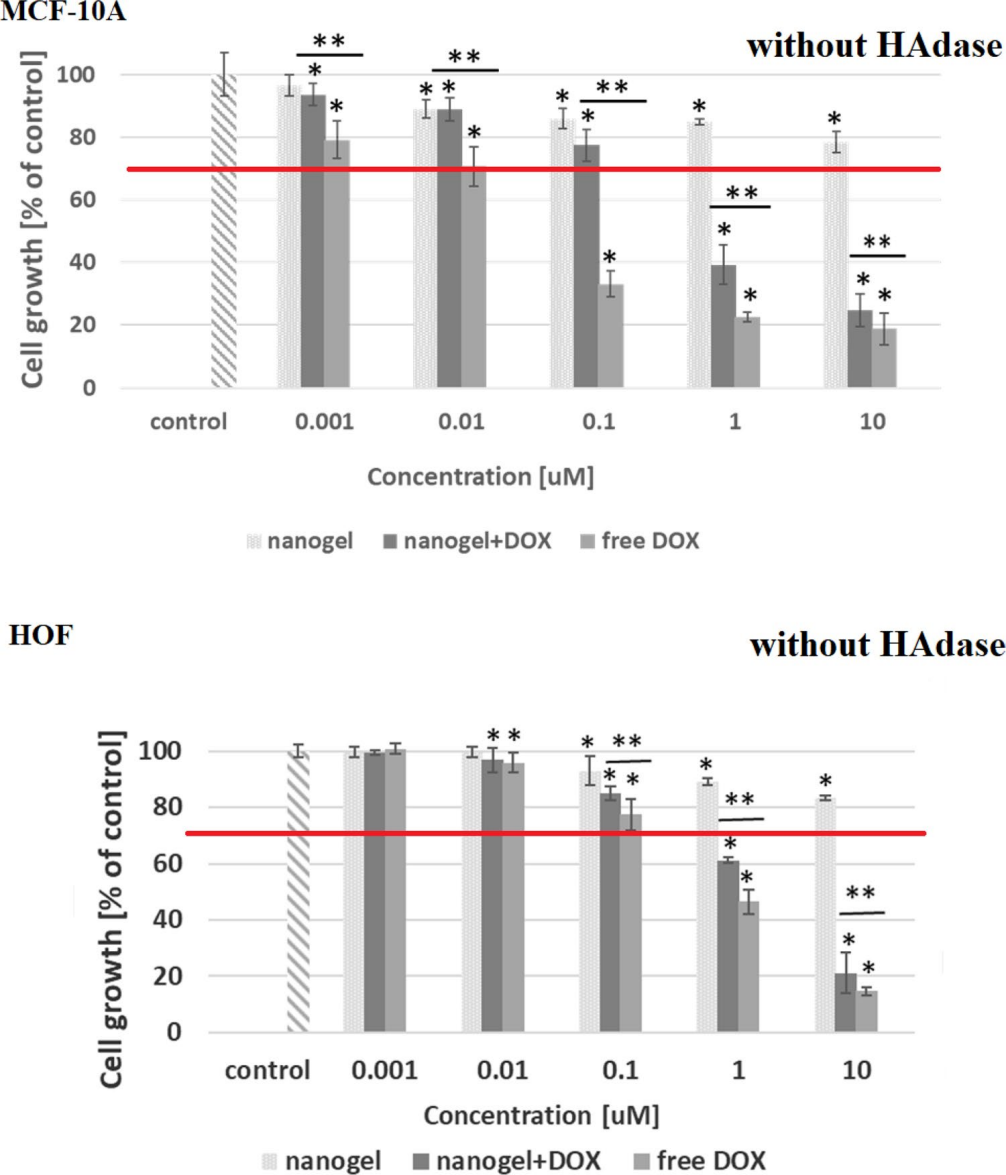
Results of MTT assay for MCF-10A and HOF healthy cell lines after 72-h treatment with free DOX, DOX-loaded and drug-free NGs. The red line depicts the threshold of 70% of cell viability in accordance with ISO 10993–5 (ISO 10993–5:2009, 2009). One way ANOVA was used to test for statistical significance. Differences from control sample were marked with *, whereas ** marked differences between groups. The difference was considered significant for P values <05.

**Figure 8.**
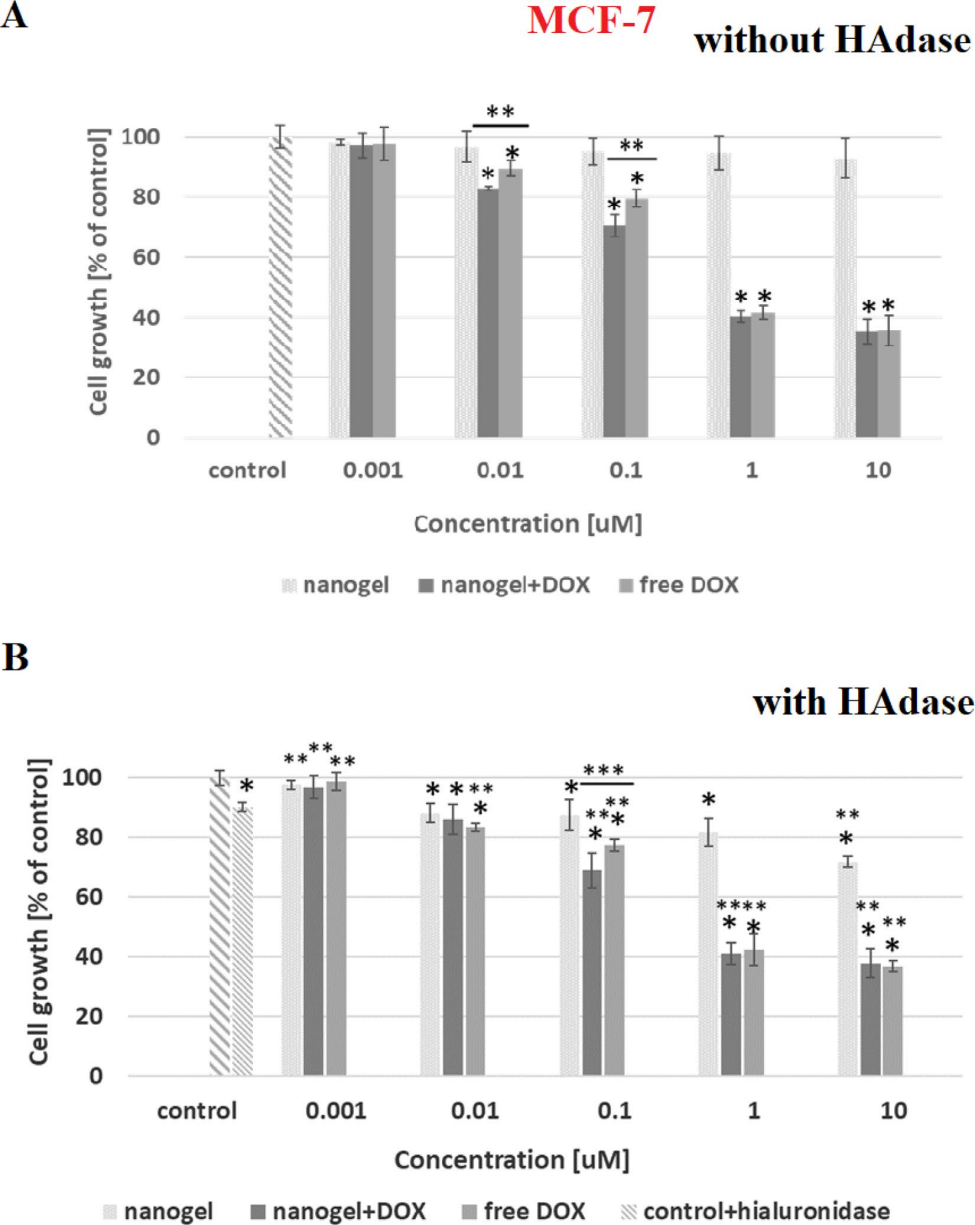
Results of MTT assay with MCF-7 cancer cell line and with and without HAdase after 72-h treatment with free DOX, DOX-loaded and drug-free NGs. One way ANOVA was used to test for statistical significance. Differences from control sample were marked with *, whereas ** marked differences between groups. The difference was considered significant for P values <05.

**Figure 9.**
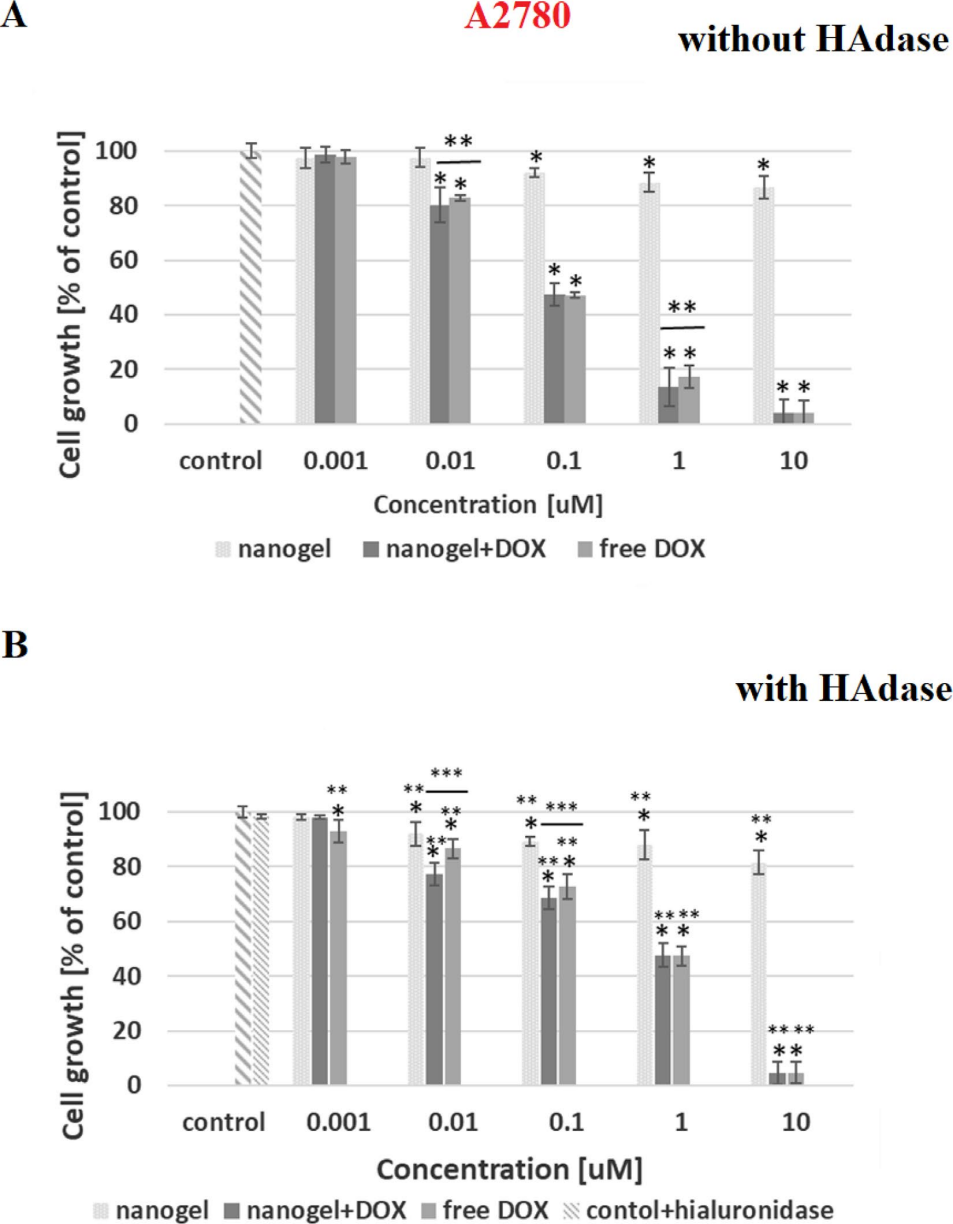
Results of MTT assay with A2780 cancer cell line and with and without HAdase after 72-h treatment with free DOX, DOX-loaded and drug-free NGs. One way ANOVA was used to test for statistical significance. Differences from control sample were marked with *, whereas ** marked differences between groups. The difference was considered significant for P values <05.

**Figure 10.**
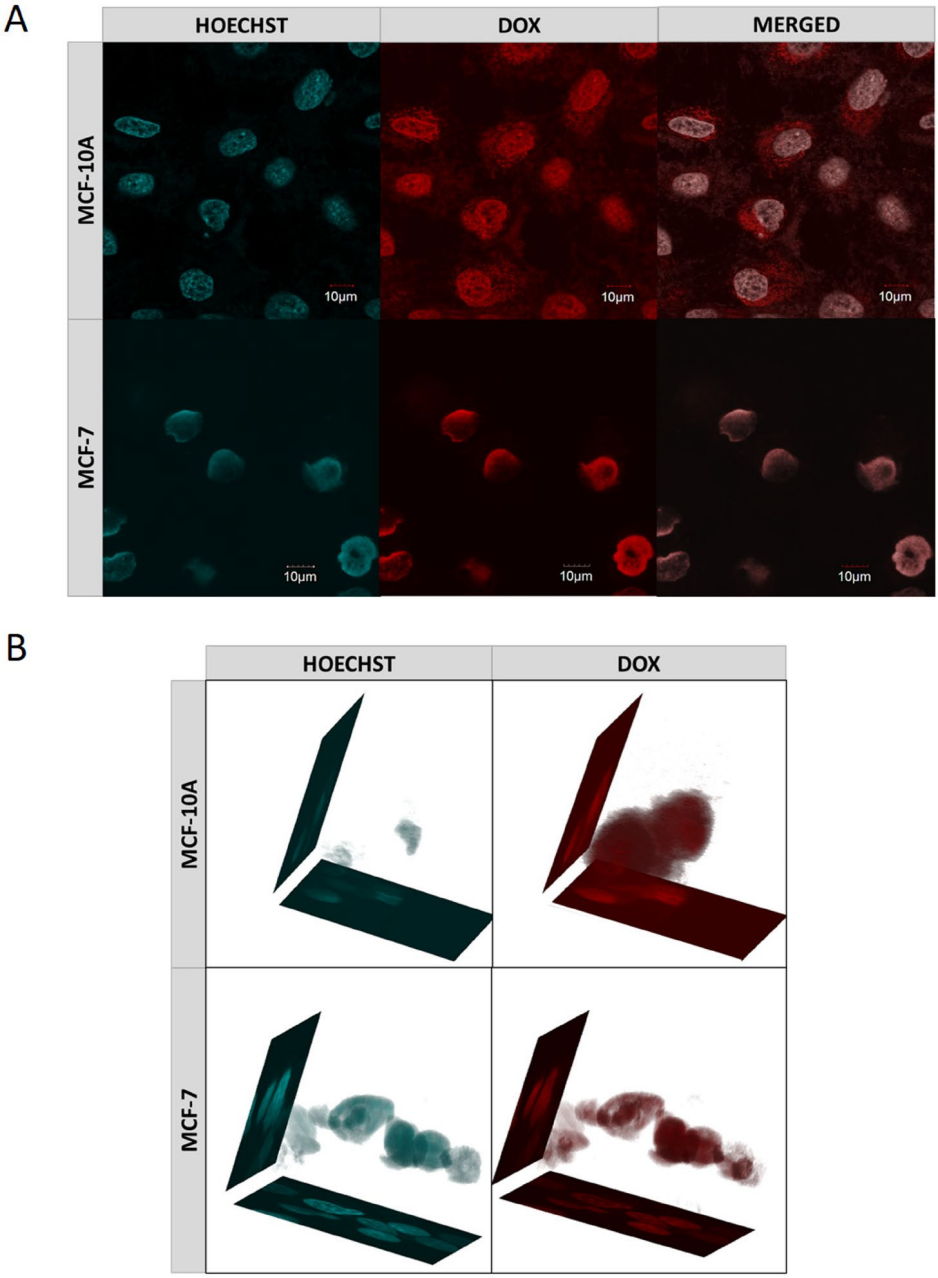
(a) Confocal images of cancer- (MCF-7) and health- (MCF-10A) cells obtained after 72-h incubation with DOX-loaded MEO_2_MA-OEGMA30%-MeHa-DEGDA NGs. Cell nuclei were stained with Hoechst fluorescent dye (blue color). Red color was emitted by DOX absorbed by cells. In case of cancer cells overlapping fluorescence signals of DOX and Hoechst dye were separated. (b) 3 D projections of MCF-7 and MCF-10A cell nuclei recorded after 72-h treatment with DOX-loaded MEO_2_MA-OEGMA30%-MeHa-DEGDA NGs. For each cell line separated fluorescent signals and merged signal are presented. Blue color marks cell nuclei stained with Hoechst dye. Red color indicates presence of DOX. (For interpretation of the references to color in this figure legend, the reader is referred to the web version of this article).

In Figures 7–10 the dependencies of viability of MCF-10A, MCF-7, HOF and A2780 cell lines on concentration of tested compounds are shown. In addition, the influence of hyaluronidase to the cytotoxicity of tested compound was examined. The obtained results showed that the free nanogel were nontoxic against cancer and healthy cells in the entire range of tested concentrations (the percentage of viability was higher than 80%). Such high value of cell growth indicates a good biocompatibility of the nanogel. For free drug and drug loaded nanogel, a decrease in cell viability with increasing drug concentration was registered. For the MCF-7 cells the IC_50_ values equaled 0.43 and 0.35 µM for free DOX and DOX-loaded nanogel, respectively. All IC_50_ values are presented in Table 4. In the case of MCF-7, additionally treated with hyaluronidase, the cytotoxicity of the drug loaded NPs was drastically increased; IC_50_ equaled 0.28 µM. In the case of healthy MCF-10A cells for both experiments, with and without addition of enzyme, the use of the nanogel as the carrier for the drug protected the cells from the DOX activity. In case of HOF healthy cells also the protective character. After an addition of HAdase we observed a slight decrease of IC50 values for MCF-10A and circa 50% decrease of HOF cells that can indicate selective sensitiveness of the cells for released DOX from degraded NGs. It should be stressed that HOF and A2780 cell lines are specifically sensitive for an interaction with DOX drug (Amarsy et al., 2022). Furthermore, for MCF-7 cells the presence of hyaluronidase increased cytotoxicity of tested compounds. The obtained results indicated that the new nanogel improved the effectiveness of cancer treatment with DOX and therefore a lower concentration of the drug will be required. In case of A2780 cells, similar, and low IC50 results for cell lines with and without HAdase indicated fast cytotoxic effect. A comparison of free drug and DOX-loaded NGs indicate that the drug effectively electrostatically interacted with HA-based NGs shells. In general, at the same time a high protection of the healthy cells was achieved.

**Table 4.** IC_50_ values for MEO_2_MA-OEGMA30%-MeHa-DEGDA NGs with or without hyaluronidase enzyme for selected cell lines.

Cell line	IC_50_ values (μM)		
**without hyaluronidase**			
		**DOX**	**NGs + DOX**
**MCF10-A**		0.04	0.51
**MCF-7**		0.43	0.35
**A2780**		0.099	0.086
**HOF**		0.77	2.78
**with hyaluronidase**			
**MCF10-A**		0.07	0.30
**MCF-7**		0.41	0.28
**A2780**		0.14	0.12
**HOF**		0.61	1.06

The influence of the nanogel on effectiveness of cell penetration by the drug was examined using a confocal microscope. Figure 9A shows MCF-7 and MCF-10A cells after 72 h of incubation with the DOX-loaded nanogel. The nuclei of the cells that were previously treated with the Hoechst dye emitted cyan light, while DOX emitted red light. A 3 D projection of the cell nuclei was also prepared, see Figure 9B. In both experiments, the drug accumulated mainly in the nuclei, whereas in the healthy cells DOX molecules are also present in the cytoplasm.

**Figure 11.**
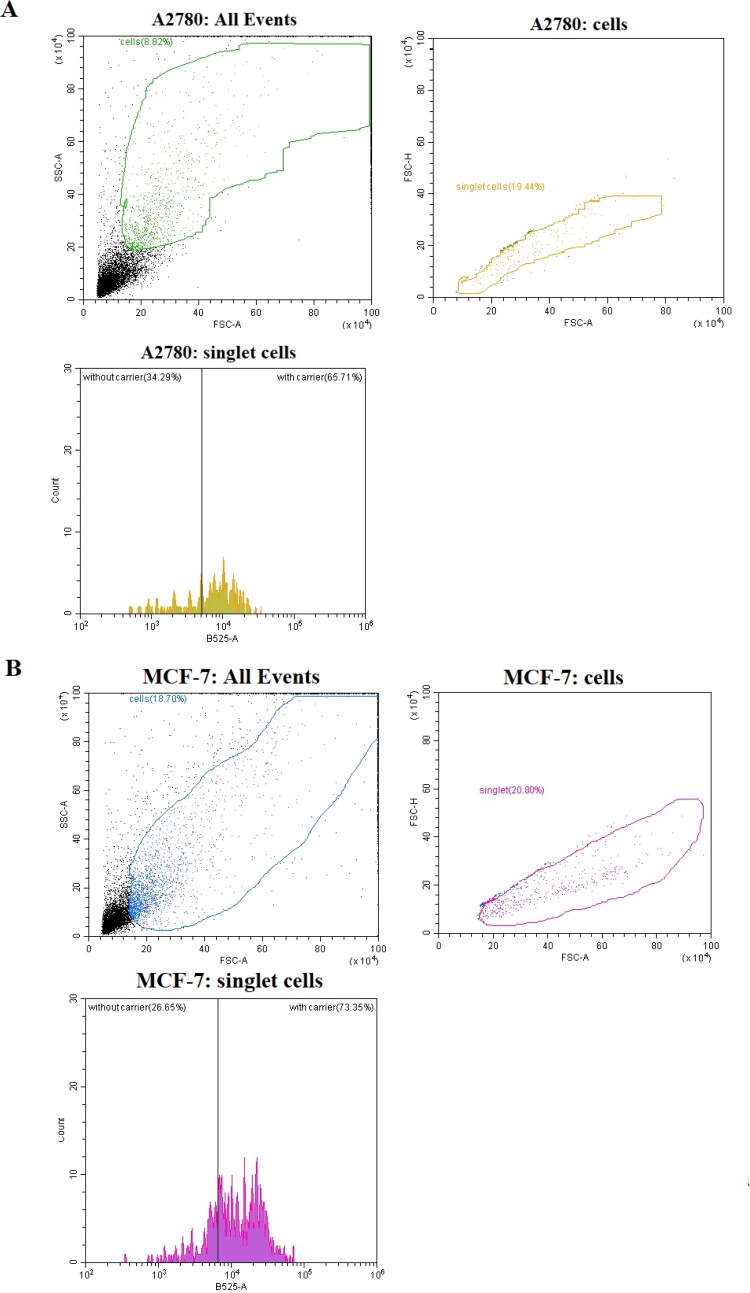
Representative flow cytometry traces characterizing A278 and MCF-7 cell lines after 24 h of incubation of NGs loaded with 0.005 µM DOX.

The flow cytometry results for 24-hour incubation of DOX-loaded NGs at levels of DOX below of the typical noticed cytotoxicity (lower that IC50 values; see Figure 11) exhibited significantly higher, from 40% to 70%, entry of DOX-loaded NGS to both types of cancer cell lines (MCF-7, A2780) compared to free drug, respectively. It should be noticed that much lower level was noticed for A2780 cell lines that not possess overexpressed typical cellular receptors (CD44, RHAMM) that are characteristic for the interaction of HA (Klapdor et al., 2021). The increased level of NGs that entered the MCF-7 suggested that targeted behavior of proposed NGs was enhanced.

## 4. Conclusions

A novel type of environmentally sensitive hybrid network MEO_2_MA-OEGMA-MeHa- DEGDA NGs containing enzyme-sensitive groups was successfully prepared by a simple precipitation polymerization. The crosslinks in the nanogels could be broken in a controlled way in the action of hyaluronidase enzyme (HAdase). The hybrid network helped to get the active targeting role to the short-lived hyaluronic acid. H^1^ NMR results confirmed an effective polymerization extent of both networks. Moreover, the electron microscopy and DLS results exhibited NPs’ tunable properties regarding the presentation of MeHa network’s carboxylic groups due to effective design of LCST (lower critical solution temperature) and shrinking of the MEO_2_MA-OEGMA part of the network. These promoted targeted delivery of selected anticancer drug to tumor cells. Additionally, novel nanogels possessed optimal parameters regarding their pH- and thermo- sensitivity, as well as aggregation, drug loading- and releasing properties, also with regard to usual hyperthermia conditions. When they were used as drug carriers of Doxorubicin, these nanogels could significantly target the tumor cells. Compared with free DOX, the DOX-loaded nanogels showed superior antitumor efficacy in breast cancer cells, MCF-7. Their activity could be controllably boosted ‘on demand’ after enriching the cell environment with an additional dose of HAdase enzyme during cytotoxicity and cellular uptake investigations. Moreover, the obtained nanogels possessed the protective role regarding the health cell lines (MCF-10A). Therefore, these targeting, biocompatible and multi-enzymatic degradable nanogels have a great potential in drug delivery systems. They can be also useful as parts of biosensing/releasing systems according to their stability in time.
